# Exploring dysfunctional barrier phenotypes associated with glaucoma using a human pluripotent stem cell-based model of the neurovascular unit

**DOI:** 10.1186/s12987-024-00593-x

**Published:** 2024-11-14

**Authors:** Sailee S. Lavekar, Jason M. Hughes, Cátia Gomes, Kang-Chieh Huang, Jade Harkin, Scott G. Canfield, Jason S. Meyer

**Affiliations:** 1 Department of Biology, Indiana University-Purdue University Indianapolis, Indianapolis, IN 46202 USA; 2 Stark Neurosciences Research Institute, Indiana University School of Medicine, Indianapolis, IN 46202 USA; 3 Department of Anatomy, Cell Biology, and Physiology, Indiana University School of Medicine, Terre Haute, IN 47809 USA; 4 Department of Medical and Molecular Genetics, Indiana University School of Medicine, Indianapolis, IN 46202 USA; 5 Department of Pharmacology and Toxicology, Indiana University School of Medicine, Indianapolis, IN 46202 USA; 6 Department of Ophthalmology, Glick Eye Institute, Indiana University School of Medicine, Indianapolis, IN 46202 USA

## Abstract

Glaucoma is a neurodegenerative disease that results in the degeneration of retinal ganglion cells (RGCs) and subsequent loss of vision. While RGCs are the primary cell type affected in glaucoma, neighboring cell types selectively modulate RGCs to maintain overall homeostasis. Among these neighboring cell types, astrocytes, microvascular endothelial cells (MVECs), and pericytes coordinate with neurons to form the neurovascular unit that provides a physical barrier to limit the passage of toxic materials from the blood into neural tissue. Previous studies have demonstrated that these barrier properties may be compromised in the progression of glaucoma, yet mechanisms by which this happens have remained incompletely understood. Thus, the goals of this study were to adapt a human pluripotent stem cell (hPSC)-based model of the neurovascular unit to the study of barrier integrity relevant to glaucoma. To achieve this, hPSCs were differentiated into the cell types that contribute to this barrier, including RGCs, astrocytes, and MVECs, then assembled into an established Transwell^®^-insert model. The ability of these cell types to contribute to an in vitro barrier model was tested for their ability to recapitulate characteristic barrier properties. Results revealed that barrier properties of MVECs were enhanced when cultured in the presence of RGCs and astrocytes compared to MVECs cultured alone. Conversely, the versatility of this system to model aspects of barrier dysfunction relevant to glaucoma was tested using an hPSC line with a glaucoma-specific Optineurin (E50K) mutation as well as a paired isogenic control, where MVECs then exhibited reduced barrier integrity. To identify factors that could result in barrier dysfunction, results revealed an increased expression of TGFβ2 in glaucoma-associated OPTN(E50K) astrocytes, indicating a potential role for TGFβ2 in disease manifestation. To test this hypothesis, we explored the ability to modulate exogenous TGFβ2 in both isogenic control and OPTN(E50K) experimental conditions. Collectively, the results of this study indicated that the repurposing of this in vitro barrier model for glaucoma reliably mimicked some aspects of barrier dysfunction, and may serve as a platform for drug discovery, as well as a powerful in vitro model to test the consequences of barrier dysfunction upon RGCs in glaucoma.

**Supplementary Information:**

The online version contains supplementary material available at 10.1186/s12987-024-00593-x.

## Introduction

Glaucoma is a complex optic neuropathy that is characterized by optic nerve degeneration and subsequent loss of retinal ganglion cells (RGCs) [1–4], leading to vision loss or blindness [5, 6]. While many risk factors exist for glaucoma [7], vasculature dysregulation is considered to be a significant risk factor [6, 8], yet mechanisms connecting vascular dysfunction and RGC damage remain unclear. At the cellular level, barriers consisting of neurons, astrocytes, and microvascular endothelial cells (MVECs), as well as contributions from pericytes and microglia [9], work together to maintain a homeostatic balance between the blood and neural tissue [10–12]. However, in several neurodegenerative diseases, these cellular barriers are compromised, resulting in the accumulation of blood-derived toxic substances within neural tissue [9, 13–16]. As such, a cellular and mechanistic basis for barrier disruption has been of considerable interest [17].

To better understand cellular contributions to barrier dysfunction associated with neurodegeneration, previous studies have established a variety of in vitro models, based primarily upon cultures of either primary or stem cell-derived MVECs [18–20]. More recent studies have incorporated other cell types of the neurovascular unit, including neurons, astrocytes, and pericytes [21–24], We have recently demonstrated that RGCs with an OPTN(E50K) mutation, causative for normal tension glaucoma [25, 26], exhibit neurodegenerative properties when differentiated from stem cells [27–29]. Additionally, we have also shown that astrocytes with this OPTN(E50K) mutation can further modulate neurodegenerative phenotypes of RGCs, conferring degenerative phenotypes even upon otherwise healthy RGCs [30], further supporting the important role for astrocytes in the neurodegenerative response [31, 32]. While previous studies have demonstrated that astrocytes with neurodegenerative mutations can compromise barrier integrity associated with other diseases [33–37], there have not been any studies to date that have leveraged similar in vitro models to analyze barrier dysfunction relevant to glaucoma. Thus, the generation of a simplified isogenic human cell-based model system mimicking certain aspects of glaucoma would provide a powerful system for understanding how interactions between cell types results in barrier dysfunction.

To address this shortcoming, the goal of our current study was to assess changes to barrier integrity by repurposing a well-established in vitro barrier model to elucidate non-cell autonomous effects in glaucoma [38–40]. To better assess possible changes relevant to the glaucoma condition, we leveraged CRISPR/Cas9-edited human pluripotent stem cells (hPSCs) with a glaucoma-associated OPTN(E50K) mutation with paired isogenic controls [27, 30]. Upon establishment of these models, barrier properties of MVECs were compromised in the presence of OPTN(E50K) RGCs and astrocytes, including decreased trans-endothelial electrical resistance (TEER) and increased permeability. Further, we identified the differential expression of TGFβ2 between isogenic control and OPTN(E50K) astrocytes, and that the modulation of TGFβ2 was able to confer or rescue barrier dysfunction properties in otherwise healthy or OPTN(E50K) conditions, respectively. Taken together, the use of this barrier model effectively mimicked certain aspects of barrier dysfunction associated with glaucoma, and successfully identified factors contributing to this dysfunctional phenotype.

## Results

### Derivation of cell types associated with barrier phenotypes

**Fig. 1:**
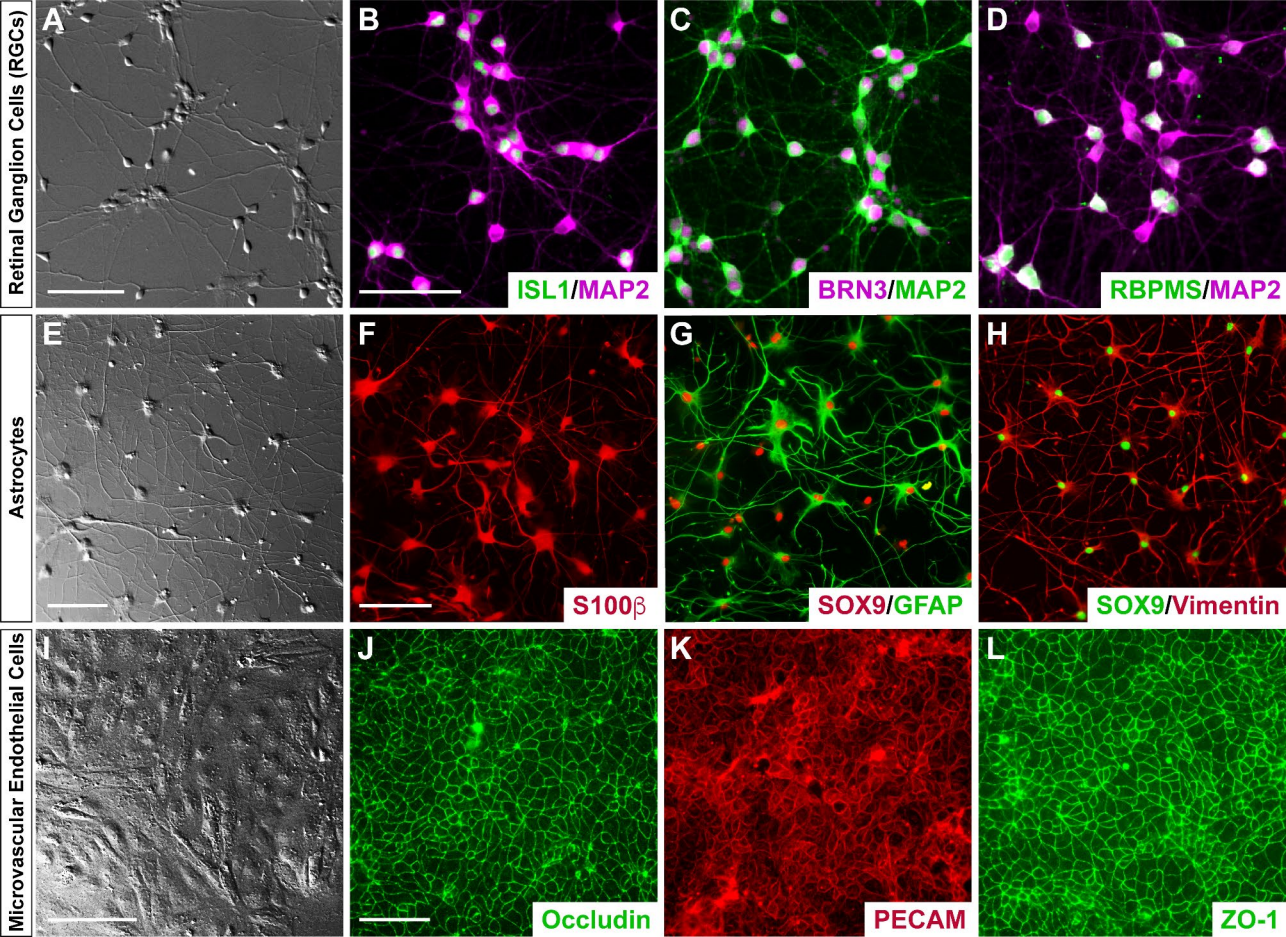
Establishment hPSC-derived RGCs, astrocytes and MVECs required for the generation of an in vitro barrier model. Human pluripotent stem cells were directed to differentiate into the different cell types needed for the in vitro barrier model, including RGCs (**A**-**D**), astrocytes (**E**-**H**), and MVECs (**I**-**L**). (**A**) DIC image of retinal ganglion cells differentiated from human pluripotent stem cell-derived retinal organoids and subsequently immunopurified by MACS. (**B**-**D**) RGCs were further characterized by immunostaining for putative RGC-specific markers BRN3, Islet1, and RBPMS, as well as cytoskeletal markers such as MAP2. (**E**) DIC image of hPSC-derived astrocytes exhibiting characteristic morphological features. (**F**-**H**) Immunostaining validated the expression of astrocyte-associated markers including S100β, GFAP, SOX9 and Vimentin. (**I**) hPSC derived MVECs validated using DIC imaging to visualize their morphological features. (**J**-**L**) Immunostaining of MVECs validated the expression of associated tight junction protein markers such as Occludin and ZO-1 along with PECAM. Scale bars equal 100 μm in **A** and **E**-**H**, and 50 μm in **B**-**D** and **J**-**L**. Scale bar in **B** applies to **C** and **D**; scale bar in **F** applies to **G** and **H**; scale bar in **J** applies to **K** and **L**

To effectively model the interactions of RGCs, astrocytes, and MVECs as an in vitro neurovascular unit, we first sought to demonstrate the effective and efficient differentiation of each cell type from cultures of hPSCs. Following differentiation, resulting cultures consisted of highly enriched populations of each cell type (Fig. 1). Differentiated RGCs were identified by the extension of lengthy neurite extensions, as well as the expression of RGC-associated markers (Fig. 1A-D), and could be highly enriched following magnetic-activated cell sorting against the Thy1.2 cell surface antigen [27, 30, 41]. Similarly, astrocytes were differentiated from hPSCs at high purity following established protocols [30, 42, 43], and these cells exhibited highly branched morphologies, as well as a variety of astrocyte-associated markers (Fig. 1E-H). Finally, MVECs were differentiated from hPSCs at high purity following established protocols [39, 40, 44], resulting in highly enriched populations of MVECs expressing a variety of markers associated with vascular endothelial cells [45–47] (Fig. 1I-L). Taken together, the results of these experiments provided sufficient evidence for the differentiation of the necessary cell types at sufficient enrichment to allow for the establishment of the neurovascular unit to study barrier integrity relevant to glaucoma.

### Co-culture of MVECs with healthy astrocytes and RGCs enhanced barrier properties

**Fig. 2:**
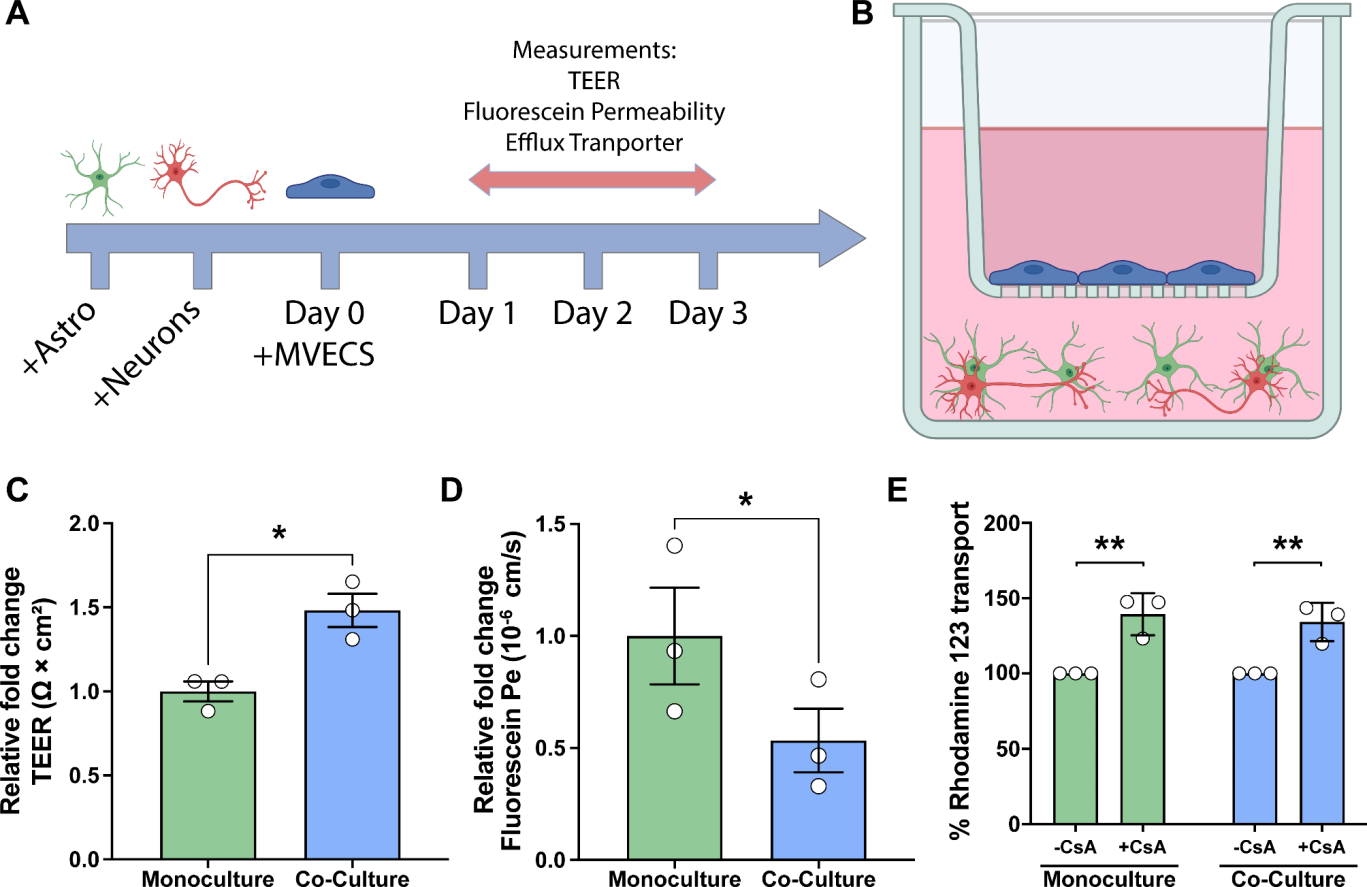
Barrier properties elevated in the healthy triple coculture model. (**A**) Schematic demonstrating the timeline used for the assembly of the triple coculture barrier transwell model. Human pluripotent stem cell (hPSC)-derived astrocytes were dissociated and allowed to mature for 3 weeks. hPSC-derived RGCs were then plated on top of astrocytes and co-cultures of astrocytes and RGCs were allowed to establish for 72 h. Subsequently, transwell inserts containing hPSC-derived microvascular endothelial cells (MVECs) were added to each well. All assays were performed at the 72 h timepoint, and MVECs grown as a monoculture were maintained as a control. (**B**) Schematic representation of the transwell model consisting of RGCs (red) and astrocytes (green) grown in the bottom of the transwell, and MVECs (blue) plated on top of the transwell inserts. (**C**) To assess barrier integrity, trans-endothelial electrical resistance (TEER) was found to be increased in cocultures compared to MVEC monocultures. (**D**) For barrier integrity analyses, paracellular transport was characterized using sodium fluorescein (10 µ; 376 Daltons), with permeability found to be considerably lower in the coculture system as compared to monocultures, suggestive of a normal functioning cellular barrier. (**E**) To test for changes in efflux activity, Rhodamine 123 transport assays demonstrated that co-culture models were equivalent to monoculture controls. Data represents mean values ± SEM from at least three independent differentiation experiments. *p = 0.0204 in **C**, ****p < 0.0001 in **D**, and **p = 0.0019 for monoculture and **p = 0.0044 for healthy cocultures in **E**. Statistical analyses included Student’s t-test in **C** and **D**, and two-way ANOVA followed by Šídák’s multiple comparisons test in **E**

Previous studies using in vitro Transwell^®^ insert barrier models have demonstrated that the most robust improvements in barrier integrity in MVECs occur due to contributions from additional neurovascular cell types [38, 48] and as such, we sought to explore whether similar findings could be obtained with differentiated RGCs and astrocytes. To this end, co-cultures of astrocytes and RGCs were assembled at a ratio of 3:1 astrocytes: RGCs, cultures of MVECs grown on inserts were added 3 days later, and assays of barrier integrity were conducted every 24 h for the next 4 days (Fig. 2A-B). Initially, the TEER as a measure of barrier integrity was analyzed. After 72 h, a timepoint previously demonstrated to exhibit maximal barrier integrity [45], MVECs grown in co-culture with RGCs and astrocytes exhibited significantly elevated TEER values compared to monoculture (Fig. 2C). Similarly, to further assess barrier integrity, the passive permeability of the barrier was assessed using sodium fluorescein, in which MVECs grown in the presence of RGCs and astrocytes exhibited a significantly lower permeability compared to MVECs grown alone (Fig. 2D). Finally, to test for changes in efflux activity, assays were performed to specifically test function of the P-glycoprotein (Pgp) efflux transporter using Rhodamine 123 as a substrate and cyclosporin-A (CsA) as Pgp inhibitor (Fig. 2E). In these experiments, while the presence of CsA significantly increased Rhodamine 123 transport in MVEC monoculture as well as in co-cultures with RGCs and astrocytes, no significant differences in efflux activity were observed when MVECs co-cultured with RGCs and astrocytes were compared to MVECs alone, suggesting that the presence of RGCs and astrocytes does not affect transcellular transport within this barrier model.

### Barrier dysfunction resulting from the OPTN(E50K) glaucoma mutation

**Fig. 3:**
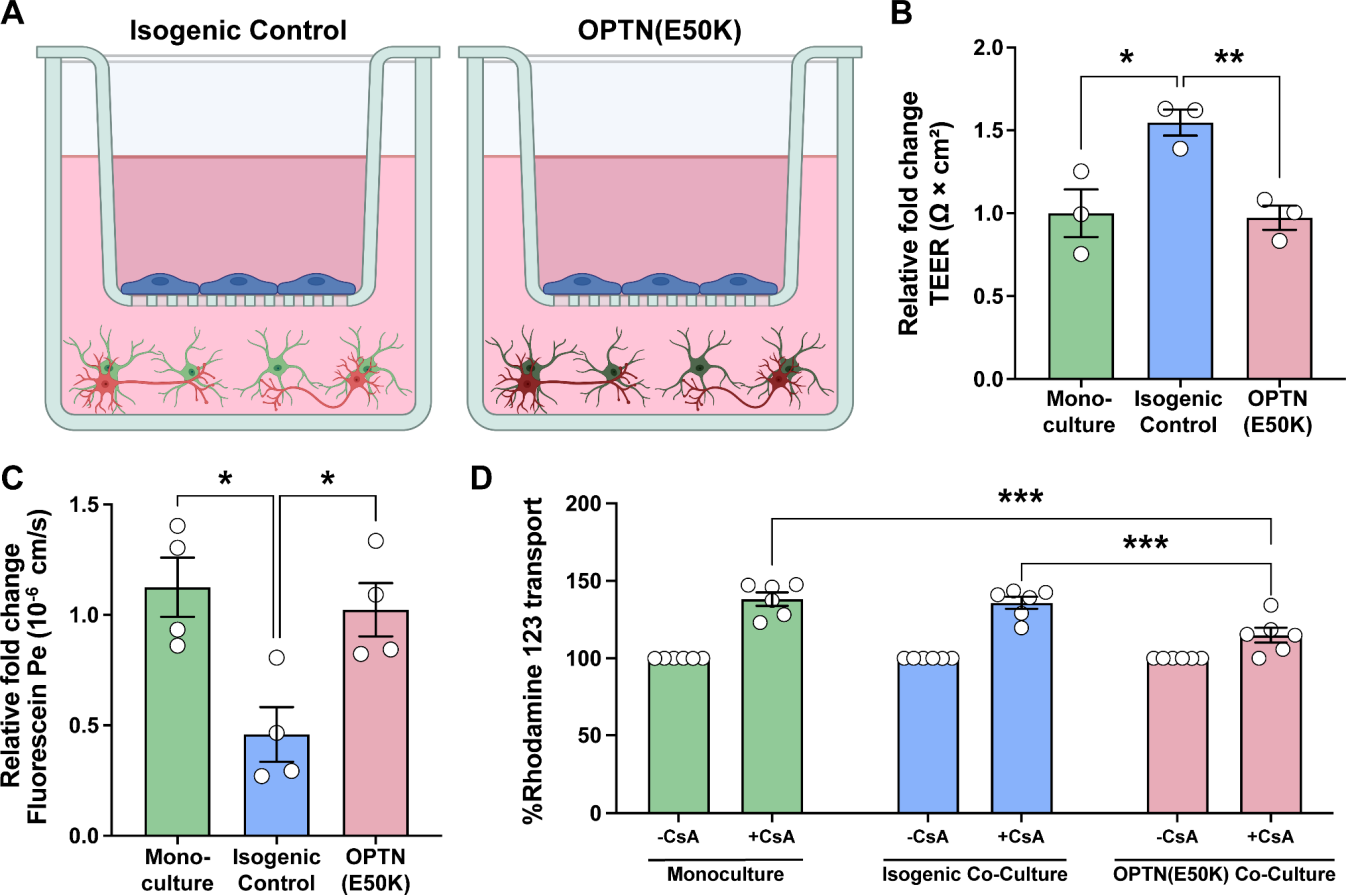
The glaucoma-associated OPTN(E50K) mutation results in impaired barrier properties. (**A**) Schematic demonstrating the experimental conditions including isogenic control RGCs and astrocytes cocultured with control MVECs in the transwell, as opposed to OPTN(E50K) RGCs and astrocytes cocultured with control MVECs. Barrier properties were assessed in comparisons between MVEC monocultures, isogenic control RGCs and astrocytes grown with MVECs, as well as OPTN(E50K) RGCs and astrocytes grown with MVECs. (**B**) Trans-endothelial electrical resistance (TEER) was significantly increased in triple co-cultures of MVECs with isogenic control RGCs and astrocytes, whereas triple co-cultures of MVECs with OPTN(E50K) RGCs and astrocytes demonstrated a decreased TEER, suggesting impaired barrier integrity. *p = 0.0121 for monoculture vs. isogenic control, **p = 0.0035 for isogenic control vs. OPTN(E50K) RGCs and astrocytes. (**C**) Paracellular transport of sodium fluorescein was significantly decreased in the triple co-cultures of MVECs with isogenic control RGCs and astrocytes compared to MVEC monocultures, while triple co-cultures of MVECs with OPTN(E50K) RGCs and astrocytes demonstrated an increased permeability. **p = 0.0096 for monoculture vs. isogenic control, **p = 0.0061 for isogenic control vs. OPTN(E50K) RGCs and astrocytes in **C**. (**D**) To test transcellular transport, Rhodamine 123 was used as a substrate along with CsA as an inhibitor for the Pgp efflux transporter. Pgp efflux transporter activity was found to be significantly decreased in triple co-cultures of MVECs with OPTN(E50K) RGCs and astrocytes, as compared to both MVEC monocultures as well as triple co-cultures of MVECs with isogenic control RGCs and astrocytes. ***p = 0.0001 for monoculture with CsA vs. OPTN(E50K) RGCs and astrocytes with CsA and ***p = 0.0006 for isogenic control with CsA vs. OPTN(E50K) RGCs and astrocytes with CsA. Data represents mean values ± SEM from at least three independent differentiation experiments. Statistical analyses included one-way ANOVA followed by Tukey’s multiple comparison test in **B** and **C**, two-way ANOVA followed by Šídák’s multiple comparisons test in **D**

As previous studies have suggested barrier dysfunction as one of several phenotypes associated with the progression of glaucomatous neurodegeneration [8, 49, 50], we then decided to assess the possibility of changes to barrier integrity as a result of the glaucoma-associated OPTN(E50K) mutation. The insert model was established using MVECs cultured directly on the insert with either healthy RGCs and astrocytes, or in the presence of RGCs and astrocytes with the OPTN(E50K) mutation cultured in the bottom chamber (Fig. 3A), and barrier phenotypes were then assessed under each condition. When comparing MVECs grown alone to those grown in the presence of healthy isogenic control RGCs and astrocytes, we again found that TEER was significantly increased in the presence of healthy RGCs and astrocytes. However, when the same experiments were performed in the presence of RGCs and astrocytes with the OPTN(E50K) mutation, we observed a significant decrease in TEER, reducing these values to approximately the same as those observed for MVECs grown alone (Fig. 3B). We then assessed passive permeability with sodium fluorescein, in which MVECs co-cultured with healthy isogenic control RGCs and astrocytes exhibited a significantly reduced permeability. However, when MVECs were co-cultured with OPTN(E50K) RGCs and astrocytes, fluorescein permeability was increased to levels similar to MVECs in monoculture (Fig. 3C). Taken together, these results suggested that RGCs and astrocytes with the OPTN(E50K) mutation did not confer any additional barrier tightening when compared to MVECs in monoculture and are significantly “leakier” compared to MVECs in co-culture with healthy RGCs and astrocytes. To further explore barrier changes due to the OPTN(E50K) mutation, we then analyzed efflux activity in MVECs in each experimental condition. We again utilized the Pgp substrate Rhodamine 1,2,3 with and without CsA to determine efflux activity, in which the presence of CsA significantly increase efflux activity in all conditions. Interestingly, while there was no significant difference observed in the presence of CsA between MVECs in monoculture and those co-cultured in the presence of healthy isogenic RGCs and astrocytes, efflux activity was significantly reduced in MVECs co-cultured with OPTN(E50K) RGCs and astrocytes (Fig. 3D). These results suggest that OPTN(E50K) RGCs and astrocytes can induce further barrier dysfunction upon MVECs via altered Pgp efflux activity.

**Fig. 4:**
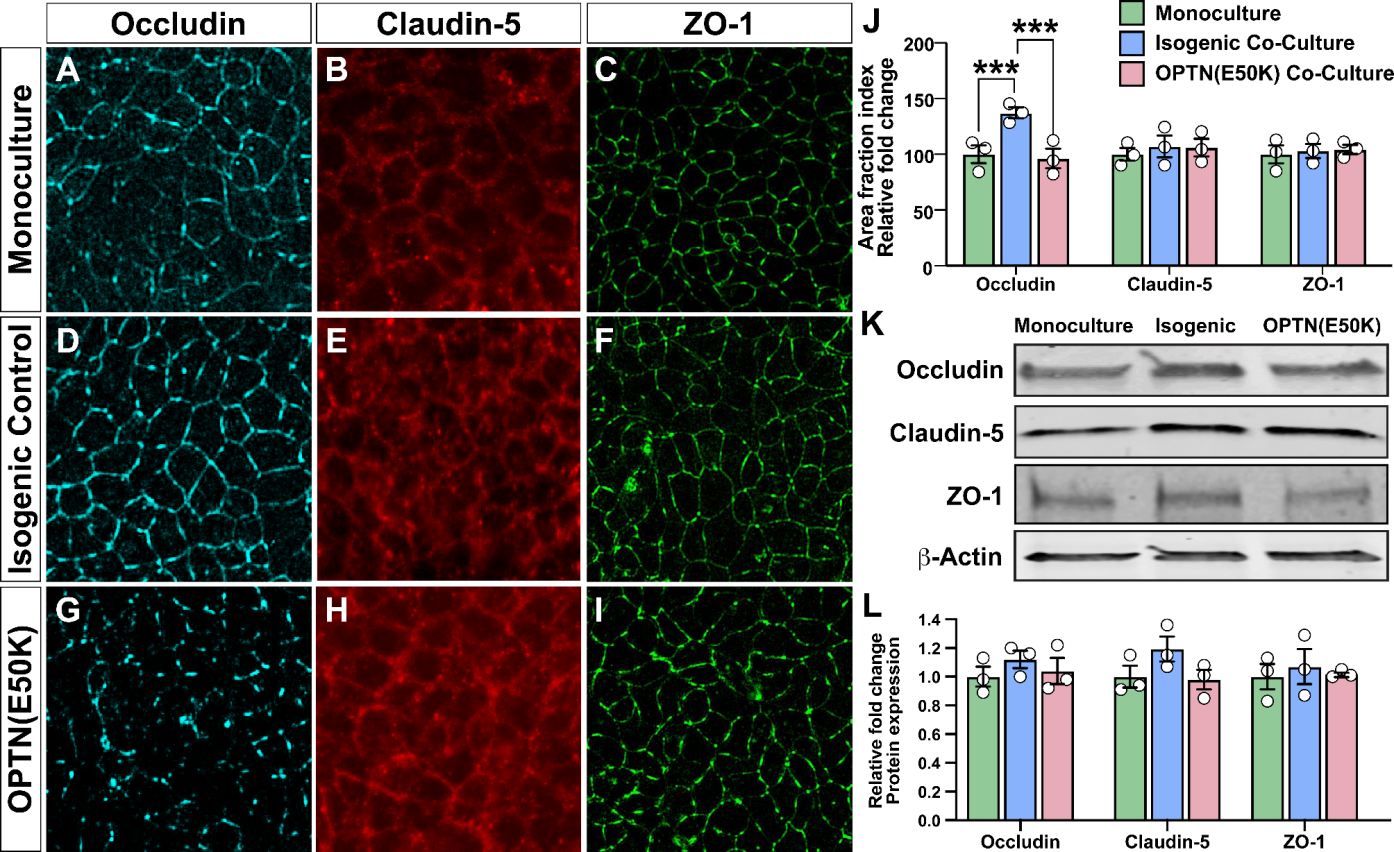
Altered localization of Occludin protein expression in MVECs co-cultured with OPTN(E50K) RGCs and astrocytes. (**A**-**I**) Representative images of immunostaining of MVEC monocultures (**A**-**C**), triple co-cultures of MVECs with isogenic control RGCs and astrocytes (**D**-**F**), or triple co-cultures of MVECs with OPTN(E50K) RGCs and astrocytes (**G**-**I**) for Occludin, Claudin-5, and ZO-1. (**J**) A comparison of tight junction protein continuity was quantified by measuring the area fraction index, in which a significant increase was observed in continuous junctions in Occludin expression in triple co-cultures of MVECs with isogenic control RGCs and astrocytes compared to MVEC monocultures or triple co-cultures of MVECs with OPTN(E50K) RGCs and astrocytes. No significant differences were observed in the localization of other tight junction proteins such as Claudin-5 and ZO-1. ***p = 0.0010 for monocullture vs. isogenic control, ***p = 0.0003 for isogenic control vs. OPTN (E50K) RGCs and astrocytes. (**K**-**L**) No significant alterations were observed in the overall expression of tight junctions analyzed by western blot. Data represents mean values ± SEM from at least three independent differentiation experiments. Statistical analyses included two-way ANOVA followed by Tukey’s multiple comparison test in **J** and **L**. Scale bar represents 100 μm

To further explore an underlying basis for at least some of these phenotypes associated with barrier dysfunction, we then explored differences in the expression or localization of characteristic tight junction proteins. Localized tight junction proteins are critical in maintaining the barrier integrity by regulating paracellular permeability [51, 52] and as such, we explored whether the localization of these proteins was altered in MVECs across these experimental groups. In these experiments, we found that the localization of Occludin was significantly increased to the peripheral edges of MVECs co-cultured in the presence of healthy isogenic control RGCs and astrocytes, but not with OPTN(E50K) RGCs and astrocytes, compared to MVEC monocultures (Fig. 4A-J). Conversely, no significant differences were observed in the localization of either Claudin-5 nor ZO-1. Western blots of cell lysates further indicated that there were no significant differences in the overall expression of tight junction proteins between MVECs grown alone or in the presence of either healthy isogenic control or OPTN(E50K) RGCs and astrocytes (Fig. 4K-L). Taken together, these results indicate, at least in part, that deficits in Occludin localization may contribute to differences observed between the experimental models.

### Identification of soluble factors contributing to barrier dysfunction

**Fig. 5:**
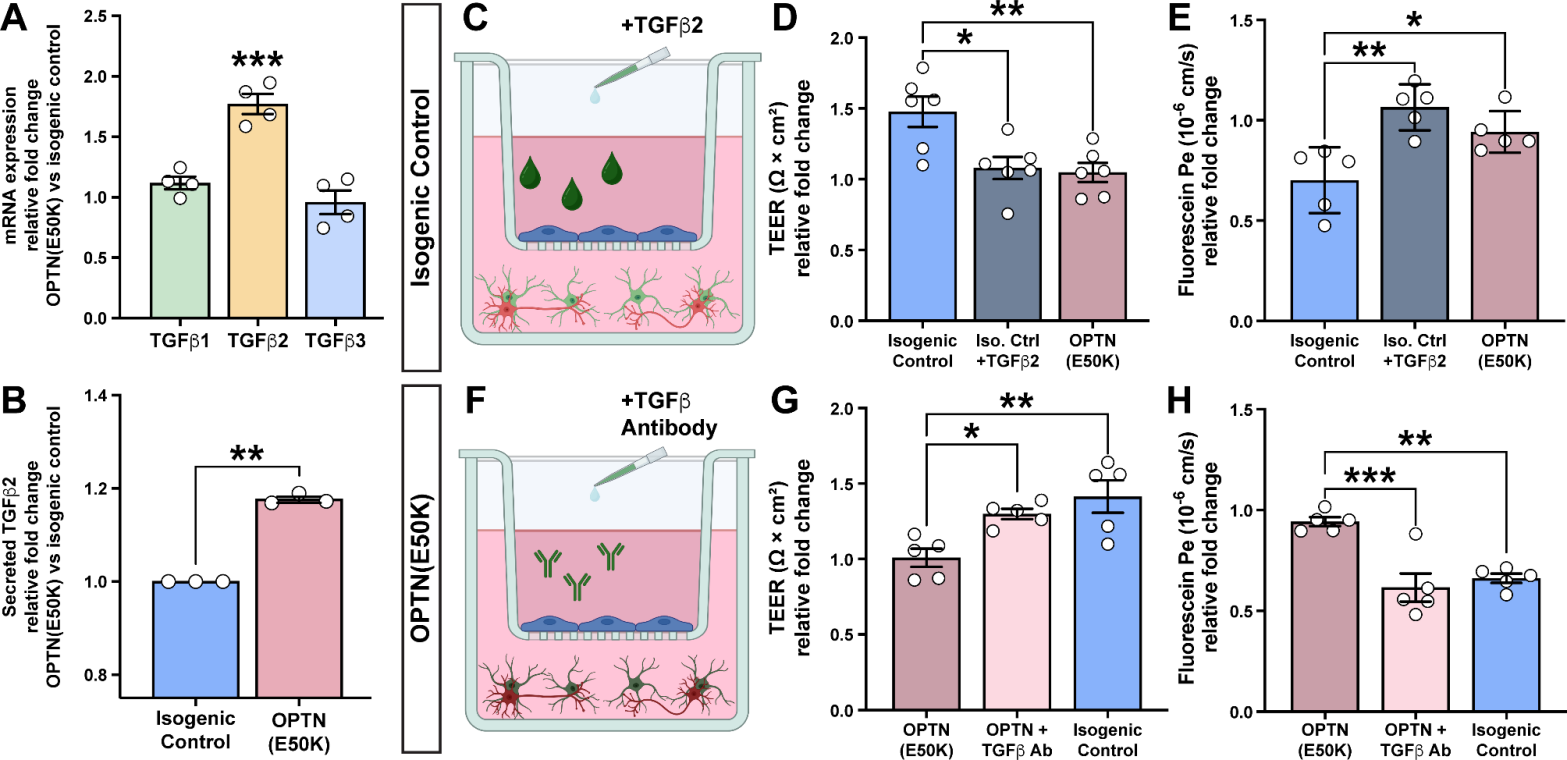
Identification of TGFβ2 as a candidate molecule to modulate barrier integrity. (**A**) A specific and significant increase in the transcriptional expression of TGFβ2 in OPTN(E50K) astrocytes compared to isogenic control astrocytes was observed by analyzing RNA-seq data (GSE173129), while no significant differences were observed in the expression of TGFβ1 or TGFβ3. Student’s t-test determined ***p < 0.001. (**B**) At the protein level, OPTN(E50K) astrocytes secreted increased levels of TGFβ2 compared to isogenic control astrocytes, as determined by ELISA. Student’s t-test determined **p = 0.0026. (**C**) Schematic demonstrating the experimental culture system consisting of isogenic control RGCs and astrocytes cocultured with MVECs in the transwell insert model. The exogenous addition of TGFβ2 to triple cocultures of MVECs with isogenic control RGCs and astrocytes resulted in a significantly decreased TEER (**D**) and increased sodium fluorescein permeability (**E**), mimicking trends observed for OPTN(E50K) RGCs and astrocytes. (**F**) Schematic demonstrating the experimental culture system consisting of MVECs co-cultured with OPTN(E50K) astrocytes and RGCs. The exogenous addition of TGFβ neutralizing antibodies led to a significant increase in TEER (**G**) as well as a significant decreased in sodium fluorescein permeability (**H**) in triple co-cultures of MVECs with OPTN(E50K) RGCs and astrocytes, mimicking trends observed for isogenic control RGCs and astrocytes. Data represents mean values ± SEM from at least five independent differentiation experiments. *p < 0.05, **p < 0.01 and ***p < 0.001, one-way ANOVA followed by Tukey’s multiple comparison test in **A**, **D**, **E**, **G** and **H**

Within the neurovascular unit, both contact-dependent and paracrine factors play a role in barrier integrity [48, 53]. The use of a Transwell^®^ insert model allows for simplicity and reproducibility across experimental groups with the potential for higher throughput, and also allows for the specific analysis of paracrine contributions from neural cells to barrier integrity. Given the differential responses we observed in barrier integrity in response to the co-culture of MVECs with either healthy isogenic control or OPTN(E50K) RGCs and astrocytes, we next sought to identify changes in secreted factors between these two groups that could at least partially underlie barrier dysfunction. Previous studies using similar insert models have determined that signaling by TGFβs can modulate barrier properties, particularly when secreted from astrocytes [48]. Interestingly, changes in the expression of TGFβ2 in the trabecular meshwork as well as optic nerve head have also been associated with glaucoma [54–57]. Thus, to further explore and identify factors underlying these differential responses, we explored differences in the expression of TGFβ1, TGFβ2, and TGFβ3 in healthy isogenic control and OPTN(E50K) astrocytes by analyzing previously acquired RNA-seq datasets using these cell lines (GEO Dataset, GSE173129) [30]. Interestingly, these analyses determined that while there was no significant difference in the expression of TGFβ1 nor TGFβ3 between isogenic control and OPTN(E50K) astrocytes, the expression of TGFβ2 was significantly increased in OPTN(E50K) astrocytes (Fig. 5A). To confirm that this transcriptional difference was then recapitulated at the protein level, particularly among secreted proteins, we then analyzed the expression of TGFβ2 by ELISA from conditioned medium of isogenic control and OPTN(E50K) astrocytes. In these studies, we found that levels of secreted TGFβ2 were significantly higher from OPTN(E50K) astrocytes (Fig. 5B), further suggesting that this secreted factor may play a role in the differential response of MVECs to isogenic control or OPTN(E50K) astrocytes.

To further test the ability of TGFβ2 to influence barrier phenotypes in MVECs in these insert models, we next sought to explore if the application of exogenous TGFβ2 to healthy isogenic control cultures could recapitulate the effects observed in response to OPTN(E50K) cells (Fig. 5C). While the co-culture of MVECs with isogenic control RGCs and astrocytes increased TEER compared to MVECs in monoculture, the treatment of this model with TGFβ2 resulted in a significantly decreased TEER response, in line with MVECs in monoculture (Fig. 5D). Similarly, while MVECs cultured in the presence of healthy isogenic control RGCs and astrocytes exhibited a reduced fluorescein permeability, the treatment of this model with exogenous TGFβ2 significantly increased fluorescein permeability (Fig. 5E). These experiments demonstrated that the addition of exogenous TGFβ2 to otherwise healthy cultures could replicate at least some of the barrier dysfunction phenotypes observed due to OPTN(E50K) RGCs and astrocytes.

Finally, we sought to test the converse situation, namely whether it was possible to block TGFβ signaling to rescue some of the barrier dysfunction phenotypes observed in the MVEC cultures grown in the presence of OPTN(E50K) RGCs and astrocytes (Fig. 5F). Compared to MVECs grown alone, the growth of MVECs in the presence of OPTN(E50K) RGCs and astrocytes once again yielded no significant changes to the TEER of this barrier. However, when neutralizing antibodies against TGFβ were added to the culture medium of MVECs in co-culture with OPTN(E50K) RGCs and astrocytes, the TEER values were significantly elevated, demonstrating tighter barrier formation (Fig. 5G). Similarly, while there was no observed difference in fluorescein permeability between MVECs grown alone and MVECs grown in the presence of OPTN(E50K) RGCs and astrocytes, the addition of the TGFβ antibodies to cultures of MVECs with OPTN(E50K) RGCs and astrocytes significantly reduced fluorescein permeability, further demonstrating increased barrier integrity (Fig. 5H). Taken together, these experiments established the ability to rescue at least some of the dysfunctional barrier phenotypes induced by OPTN(E50K) RGCs and astrocytes through modulation of TGFβ signaling.

## Discussion

The results of this study document the use of an established in vitro model of the neurovascular unit to examine features resulting in barrier dysfunction associated with glaucoma using hPSCs with an OPTN(E50K) mutation. As a neurodegenerative disease, glaucoma is characterized by a prominent loss of RGCs with an eventual loss of vision. We have previously established that hPSC-derived RGCs with the glaucoma-associated OPTN(E50K) mutation exhibit morphological and functional deficits [27, 58] and more recently, we demonstrated that astrocytes with the OPTN(E50K) mutation can confer neurodegenerative phenotypes upon otherwise healthy RGCs [30], underscoring the important role that astrocytes play in overall homeostasis. As astrocytes serve in numerous important roles, including critical contributions to the neurovascular unit, and previous studies have demonstrated that vascular dysfunction is also a characteristic of glaucoma [8, 49, 50], we sought to explore whether hPSC-derived RGCs and astrocytes with the OPTN(E50K) mutation could similarly modulate barrier properties in an established Transwell^®^ model of the neurovascular unit. Overall, we found that RGCs and astrocytes with the OPTN(E50K) mutation resulted in compromised barrier integrity, and further identified elevated levels of TGFβ2 as a candidate factor contributing to this barrier dysfunction, thereby establishing this model as a powerful tool for the study of barrier dysfunction in a model relevant to glaucoma.

The identification of TGFβ2 as a secreted factor that partially contributes to barrier dysfunction serves as a further validation of this model, including the ability to identify specific factors contributing to dysfunction. In order to serve in this capacity, it is essential to utilize isogenic pairs of hPSCs in order to minimize the effects of any other genomic differences [59, 60]. In this study, we have utilized isogenic pairs of cells that contain the OPTN(E50K) mutation along with a paired isogenic control, as previously described [27, 30]. In this way, the identification of elevated TGFβ2 associated with the OPTN(E50K) cells is due more specifically to that disease-associated mutation rather than other genomic differences between cell lines. It is of considerable interest that TGFβ2 was identified as a factor that contributes to barrier dysfunction, as previous studies have documented the elevation of TGFβ2 in the eyes of patients with glaucoma [54, 61–63]. While many of these studies have focused upon the differential regulation of TGFβ2 in cells of the trabecular meshwork, some studies have also documented elevated TGFβ2 within the optic nerve head as well [64, 65], the primary site of RGC injury and the location in which astrocytes are known to contribute to RGC neurodegeneration in inflammatory conditions. Interestingly, our efforts to modulate TGFβ2 levels was closely associated with barrier integrity or dysfunction, with exogenous TGFβ2 leading to barrier dysfunction in the healthy control model, while inhibition of TGFβ2 rescued some features of barrier dysfunction in the OPTN(E50K) model. However, this rescue was not complete, suggesting that other factors are likely to contribute to these properties, including perhaps the lack of other cell types such as pericytes in the system that modulate the barrier, along with the pivotal role of other factors missing in this in vitro model that are usually present in vivo to compensate for some features.

It was also intriguing that in these current studies, our results demonstrated that the application of exogenous TGFβ2 resulted in barrier dysfunction, whereas previous studies have shown that the application of exogenous TGFβ2 enhanced barrier integrity [48]. An important distinction between these studies is that our current results tested the effect of exogenous TGFβ2 upon cultures of MVECs in the presence of RGCs and astrocytes, whereas previous studies examined the application of TGFβ2 to monocultures of MVECs that were also derived differently in the absence of retinoic acid, resulting in lower barrier integrity. The disparity in these results suggests that the effects of TGFβ2 upon barrier permeability are likely dose-dependent, with some TGFβ2 enhancing barrier integrity and perhaps responsible for some improvements observed with healthy RGCs and astrocytes, whereas even more TGFβ2 may be detrimental. Further, it suggests that differences in MVECs may also affect their response to exogenous factors such as TGFβ2. In future studies, it will also be of interest to look for more candidate compounds associated with barrier dysfunction, as well as observe the impact of factors known to be influential in the neurovascular unit, such as VEGF.

In this study, we utilized MVECs derived from human iPSCs that have been previously characterized as “brain-like” due to the limited ability to derive retinal MVECs from iPSCs. Brain-like MVECs express several critical barrier properties but do genetically express a mixed epithelial-endothelial lineage [66]. A current limitation in the barrier field is the inability to derive a genetically identical in vivo MVEC that also displays robust barrier phenotypes. Our current model displays several critical phenotypes as similarly observed in both primary human and animal-derived models. Furthermore, brain and retinal MVECs share many of these phenotypes, however, their expression profiles can vary [67], and thus could affect these study’s findings. As more robust human iPSC-derived retinal MVECs with in vivo-like expression profiles and phenotypes become established, their incorporation into the co-culture model will certainly advance the field.

While the emphasis of this study was upon the effects of RGCs and astrocytes with the glaucoma-associated OPTN(E50K) mutation upon barrier-forming MVECs, in future studies it will be of interest to explore the impact of a dysfunctional barrier upon RGCs and astrocytes. It is possible that MVECs differentially regulate neural cells based upon their degree of barrier integrity, or perhaps MVECs with a disease-associated mutation may contribute to neurodegeneration. Additionally, the use of this type of model may also allow for the study of barrier permeability and the effects of various cell types upon the overall health of neural cells. For simplicity purposes, our current model also utilized iPSC-derived RGCs and astrocytes in co-culture with MVECs to further elucidate the role of glaucoma-induced factors in barrier dysfunction. Future studies will expand upon the co-culture model to incorporate pericytes and microglia as we pursue additional glaucoma-induced mechanisms in barrier dysfunction. Various combinations can be pursued for the different cell types with and without the mutation to identify potential triggering points of impact that eventually lead to neurodegenerative phenotypes, essentially to identify the cause and effect of the underlying mutation.

In the current studies, changes in barrier permeability were associated at least in part with changes in the expression of tight junction proteins such as Occludin. Interestingly, no changes were observed in the overall expression of either Occludin, Claudin-5, nor ZO-1, yet changes were found specifically within the cellular localization of the Occludin protein, with MVECs grown in co-culture with OPTN(E50K) RGCs and astrocytes exhibiting a significant increase in the percentage of discontinuous junctions for Occludin compared to those grown with isogenic control RGCs and astrocytes. Previous studies have shown that tight junction protein dysfunction can be either dependent on one critical tight junction protein being affected or multiple [35, 53, 68, 69]. This can be explained by a potential coping mechanism of the system which demonstrates modulation of only one tight junction protein which is concomitantly equilibrated by the other TJPs in certain neurodegenerative diseases.

In the interpretation of these results, it is also important to note the timeframe in which effects were observed. Consistent with previous studies using similar cellular insert models [44, 46], peak barrier integrity typically occurred 48–72 h following the establishment of the barrier model, with decreases in barrier integrity beginning after that even within the otherwise healthy model. Within this timeframe, we were readily able to observe significant decreases in barrier integrity due to the OPTN(E50K) mutation. However, since the barrier integrity decreases after time, the model is likely better for analyses of acute changes rather than chronic conditions. The limited timeframe for the utility of this model is likely due to a variety of factors, including the lack of cell types that are essential for the maturation of the barrier such as pericytes and microglia. More recently, some studies have demonstrated that an initial priming of MVECs by pericytes allow for a modest degree of further MVEC maturation [21, 22, 70]. Thus, in future studies, it may be helpful to incorporate pericytes into this model. Furthermore, the cellular insert model allows only for the study of paracrine effects upon barrier integrity. While this provides a significant strength in that experiments can be performed in a more high-throughput and more highly reproducible manner, future studies can explore more advanced models of the neurovascular unit that take cell-cell contact into consideration.

## Methods

### Maintenance of human pluripotent stem cells

Cell lines used in this study included the H7 embryonic stem cell line [71] as well as the IMR90-4 iPS cell line [72]. hPSCs were expanded and passaged either with dispase or versene approximately every 6 days at a 1:6 ratio, and grown on a Matrigel substrate in mTeSR1 medium with daily media changes, as previously described [73, 74]. For the differentiation of RGCs, we used lines that were previously edited to express a BRN3b: tdTomato: Thy1.2 transgenic reporter for the identification and purification of RGCs [27, 30, 41]. Further, for some experiments, we used a line that was further edited to harbor the OPTN-E50K mutation, as previously described [27].

### Differentiation of hPSCs into RGCs, astrocytes and MVECs

RGCs were differentiated through the initial formation of retinal organoids, as previously described [75–77], and subsequent isolation and purification of RGCs. Initially, colonies of hPSCs were lifted by enzymatic dissociation using dispase (2 mg/ml) to form embryoid bodies (EBs), followed by slowly transitioning from mTeSR1 medium to a neural induction medium (NIM; DMEM/F12, 1x N2 supplement, MEM non-essential amino acids, and heparin (2 ug/ml)) over a period of 3 days. To induce a retinal lineage, BMP4 (50 ng/ml) was added at day 6 of differentiation, and EBs were then induced to adhere to the culture plate on day 8 using 10% fetal bovine serum (FBS). BMP4 was then reduced every 3 days to half the concentration with half media changes, until Day 15 when a full media change was performed. On Day 16, early retinal organoids were then mechanically lifted from the plate and transitioned into a retinal differentiation medium (RDM; DMEM/F12 (3:1), 1x B27 supplement, and anti-anti). Retinal organoids were then maintained in RDM with media changes every 2–3 days. On Day 45, retinal organoids were enzymatically dissociated using accutase, and RGCs were purified by magnetic activated cell sorting based upon expression of the Thy1.2 cell surface antigen, as previously described [30, 41].

Astrocytes were differentiated from hPSCs using modifications of the above protocol as previously described [30, 42, 78], with the exception that on day 6 of differentiation, BMP4 was excluded to prevent the induction of a retinal lineage in favor of a forebrain neural fate. To further induce glial differentiation, aggregates of neural progenitor cells were expanded in the presence of FGF2 (20 ng/ml), EGF (20 ng/ml), and heparin (2 ug/ml) in RDM, and aggregates were mechanically chopped to smaller clusters approximately every 2 weeks using a McIlwain tissue chopper to prevent necrosis of the core of the aggregates. After a total of at least 6 months of differentiation, cell aggregates were enzymatically dissociated with Accutase, and astrocyte progenitors were then plated in BrainPhys medium for 3 weeks for maturation.

To yield MVECs, hPSCs were differentiated using an established 10-day protocol with daily mTESR medium changes, as previously described [22, 38]. Briefly, hPSCs were singularized using accutase and re-plated at 30,000 cells/cm^2^ followed by maintenance in unconditioned medium (UM), consisting of DMEM/F12 with 0.1 mM beta-mercaptoethanol and 1x MEM non-essential amino acids, for 5 days to initiate cell differentiation. On Day 6, UM is changed to human Endothelial Serum-Free Medium (EC +/+) supplemented with bFGF (20 ng/ml), 1% platelet-poor plasma-derived bovine serum and 10 µM retinoic acid. At this point, MVECs were isolated and plated for experimental approaches in Endothelial Serum-Free Medium lacking bFGF (EC +/-).

### Establishment of Transwell® barrier model

To establish the Transwell^®^ barrier model, MVECs were differentiated and then dissociated with accutase and re-plated onto inserts coated with collagen/fibronectin/water (ratio of 1:4:5) at a density of 1 million cells/cm^2^. In parallel, astrocytes were dissociated followed by plating onto laminin-coated 12 well plates and allowed to mature for 3 weeks. Subsequently, RGCs were purified from retinal organoids using Thy1.2 microbeads via magnetic activated cell sorting and plated on top of 3-week-old astrocytes at a ratio of 1:3 RGCs: astrocytes. Cocultures of RGCs and astrocytes were allowed to establish for 72 h, followed by the addition of transwells containing MVECs. In control experiments, MVECs were maintained as a monoculture grown alone.

### Immunocytochemical analyses

Immunostaining was performed using protocols as previously described [79, 80]. Briefly, cells were fixed using 4% paraformaldehyde or ice cold methanol for 30 min, followed by 3x washes with 1X PBS for 5 min each. Cells were then permeabilized with 0.2% Triton X-100 for 10 min immediately followed by a 1x PBS wash. Cells were then blocked with 10% donkey serum for 1 h followed by primary antibody overnight in 0.1% Triton X-100 and 5% donkey serum at 4 °C. Primary antibodies used are listed in Supplemental Table 1. The next day, cells were washed 3x with PBS and incubated with 10% donkey serum for 10 min. Secondary antibodies along with DAPI were applied for 1 h in 0.1% Triton X-100 and 5% donkey serum at room temperature. Lastly, cells were washed 3x with PBS, and coverslips were mounted on slides using mounting medium. For the measurement of the continuity of tight junction proteins, ImageJ software was used to measure the area fraction index. Three independent experiments were analyzed for measuring the continuity of the tight junction proteins with at least ten fields of at least 30 cells/field.

### Measurement of trans-endothelial electric resistance

Trans-endothelial electrical resistance (TEER) was recorded every 24 h following subculture of MVECs, either alone or in transwell co-cultures with RGCs and astrocytes. TEER measurements were recorded with an EVOM ohmmeter with STX2 electrodes (World Precision Insturments, Sarasota, FL, USA), following subtraction of unseeded transwells and normalized to transwell surface area. TEER values obtained were in the range of 1590–2643 Ω × cm^2^ for monoculture, 2562–3440 Ω × cm^2^ for isogenic control co-cultures, 1319–2281 Ω × cm^2^ for OPTN(E50K) co-cultures, 967–2325 Ω × cm^2^ for isogenic control co-cultures treated with TGFβ2, and 1585–3977 Ω × cm^2^ for OPTN(E50K) co-cultures treated with TGFβ2 neutralizing antibodies. To account for variability across replicated experiments, TEER measurements are represented as a fold change compared to the monoculture of MVECs. TEER measurements were recorded immediately following removal from the incubator. Resistance was measured at least three independent times for each sample with a minimum of three independent replicates for each experimental condition.

### Modulation of TGFb2 signaling within in vitro barrier model

To test the effects of TGFβ signaling within the in vitro barrier model, we either added exogenous TGFβ2 to isogenic control cultures, or inhibited TGFβ signaling in OPTN(E50K) cultures by addition of a neutralizing antibody. To stimulate isogenic control co-cultures, TGFβ2 (R&D Systems, 302-B2) was added to barrier models at a final concentration of 10 ng/ml. Conversely, to inhibit TGFβ signaling in OPTN(E50K) cultures, a neutralizing antibody to TGFβ1/2/3 (R&D Systems, MAB1835R) was added to barrier models at a final concentration of 0.05 µg/ml. TGFb signaling modulators were added 24 h post-seeding onto transwells/experimental plates and remained there for the duration of experiments to test either TEER, fluorescein permeability, or rhodamine transport.

### Measurements of sodium fluorescein permeability

Paracellular transport was assessed using sodium fluorescein (10 µM; 376 Daltons; Sigma Aldrich). Prior to the assay, MVEC transwells were removed from co-culture with RGCs/astrocytes and fresh EC +/- medium was added (0.5 mL Top / 1.5 mL Bottom). Fluorescein was then added to the apical side of the transwell, and cultures were placed on a rotational platform at 37 °C. 150 ul aliquots were taken at 15, 30, 45, and 60 min from the bottom chamber, and pre-warmed EC +/- medium replenished the volume removed for sampling at each time point. Fluorescence was recorded using a Synergy HTX Multi-Mode reader (BioTek, Vermont, USA). Permeability coefficients were calculated based on the cleared volume of fluorescein from the top chamber to the bottom chamber.

### Efflux transporter activity/ accumulation assay

Efflux transporter activity of P-glycoprotein (Pgp) was measured using Rhodamine as a Pgp substrate and Cyclosporin A (CsA) as an inhibitor for Pgp efflux transporter activity. CsA was added to cultures for 1 h at 37 °C on a rotating platform followed by addition of rhodamine. The uptake of rhodamine in MVECs was measured by collecting lysates for the measurement of rhodamine uptake using a fluorescence plate reader.

### Western blot

Cell lysates were collected from MVECs using Radioimmunoprecipitation assay buffer with phosphatase protease inhibitor cocktail or Mper protein extraction reagent. Lysates were then centrifuged at 14,000 g for 10 min and the cell pellet was collected and used for the immunoblotting assay. BCA assay was used to quantify 30 µg of protein for all samples, which was then loaded into an SDS-PAGE gradient (4–15%) gel and run at 75 V for 15 min and then 165 V for 40 min. Subsequently, the Transblot Turbo system was used for the transfer of proteins to a nitrocellulose membrane. After transfer, ponceau stain was used for visualizing the proteins on the nitrocellulose membrane, which was then washed with water after visualization and then washed with 5% milk in TBST for 3X washes for 5 min each. The primary antibody of interest (Supplemental Table 1) was then added overnight with continuous shaking at 4 degrees. The following day, the blot was washed again with 1X TBST three times for 5 min each, followed by addition of the appropriate secondary antibody using 5% blocking buffer in 1X TBST for 1 h at room temperature on the shaker. The blot was then washed 3X in TBS, and then visualized using the Li-Cor Odyssey system. Western blot data was analyzed by acquiring ratio of the protein of interest and total actin and the relative fold change value was calculated and plotted with respect to the monoculture values for the respective assays.

### Enzyme linked immunosorbent assays

To quantify the level of TGFβ2 produced by these cell cultures, conditioned medium was collected after 3 weeks of maturation, both from isogenic control cultures as well as from the OPTN(E50K) cell line. The conditioned medium was then concentrated 40x times using the Pierce Protein Concentrator kit, including centrifugation at 14,000 g at 4 °C. ELISA assays were then performed on these sample using the TGFβ2 ELISA kit (Life Technologies), with absorbance quantified using a spectrophotometer.

### Statistical analyses

All data throughout the manuscript are presented as mean ± SEM. Statistical analyses were performed using Graphpad Prism software, using one way ANOVA for multiple samples, two way ANOVA with two variables, or Student’s t-test for paired samples, as appropriate. Significance was determined in each case with a p value < 0.05.

## Electronic supplementary material

Below is the link to the electronic supplementary material.

Supplementary Material 1

## Data Availability

No datasets were generated or analysed during the current study.
