# Supplementary material for: Exploring dysfunctional barrier phenotypes associated with glaucoma using a human pluripotent stem cell-based model of the neurovascular unit
Source: Fluids Barriers CNS. 2024 Nov 14;21:90. doi: 10.1186/s12987-024-00593-x (PMC11566410; doi:10.1186/s12987-024-00593-x)
Supplement: Supplementary file 1 — Supplementary Material 1 [file 12987_2024_593_MOESM1_ESM.pdf]

Table S1. List of antibodies used in the study.

Antibody	Type	Source	Catalog	Dilution
Actin, beta	Mouse monoclonal	Sigma-Aldrich	A5441	1:10000
BRN3	Goat polyclonal	Santa Cruz Biotechnology	sc-514474	1:200
Claudin-5	Mouse monoclonal	Invitrogen	4C3C2	1:100
GFAP	Mouse monoclonal	Millipore	MAB360	1:200
ISL1	Mouse monoclonal	Developmental Studies Hybridoma Bank	40.2D6	1:200
MAP2	Mouse monoclonal	Synaptic Systems	188011	1:200
Occludin	Mouse monoclonal	Invitrogen	OC-3F10	1:50
PECAM	Rabbit Polyclonal	Lab Vision	RB-10333	1:25
RBPMS	Rabbit polyclonal	Phosphosolutions	1830-RBPMS	1:500
S100β	Mouse monoclonal	Abcam	ab218513	1:200
SOX9	Goat polyclonal	R&D Systems	AF3075	1:200
TGFβ	Mouse monoclonal	R&D Systems	MAB1835R	1:10,000
Vimentin	Mouse monoclonal	Santa Cruz Biotechnology	sc-6260	1:100
ZO-1	Mouse monoclonal	Thermo Fisher	ZO1-1A12	1:200
